# Supplementary material for: Correction of non-random mutational biases along a linear bacterial chromosome by the mismatch repair endonuclease NucS
Source: Nucleic Acids Res. 2024 Mar 6;52(9):5033–47. doi: 10.1093/nar/gkae132 (PMC11109965; doi:10.1093/nar/gkae132)
Supplement: gkae132_Supplemental_File [file gkae132_supplemental_file.pdf]

Supplementary Table S1. Primers used in this study.

Name	Sequence (5'-3')	Purpose
nucS_compl_F	AGGGGAATTCAGACGTCGAGCGACATGGGT	Cloning *nucS* in pSET152 for the creation of complemented strains
nucS_compl_R	CGAGGAATTCGACGGAGCAGATCATCTGAGC	
nucS_F	GTTGCCGCATATGCGTCTCGTCATTGCCCGCT	Cloning *nucS* in pET15b for heterologous expression
nucS_R	TGTAGGATCCTCAGAACAGCCGCAGCTTG	
dnaN_F	TAGCATATGAAGATCCGGGTGGAACGC	Cloning *dnaN* in pET15b for heterologous expression
dnaN_R	ACTGGATCCTCAGCCGCTCAGCCGCAC	

Supplementary Table S2. Sequence of oligonucleotides used for cleavage assays (left) and combination of the oligonucleotides to form heteroduplex DNA with a mismatch (right).
Asterisk means that the sequence is 6FAM labeled at 5′

Number	Sequence (5'-3')
1*	ACCCAGTACTCGCTGCTGAATGGAGCCGCGCGGCTGAAGGACA
2*	TGTCCTTCAGCCGCGCGGCTCCGTTCAGCAGCGAGTACTGGGT
3*	ACCCAGTACTCGCTGCTG
4*	TGTCCTTCAGCCGCGCGGCT
5	TGTCCTTCAGCCGCGCGGCTCCATTCAGCAGCGAGTACTGGGT
6	TGTCCTTCAGCCGCGCGGCTCCGTTCAGCAGCGAGTACTGGGT
7	TGTCCTTCAGCCGCGCGGCTCCTTTCAGCAGCGAGTACTGGGT
8	TGTCCTTCAGCCGCGCGGCTCCCTTCAGCAGCGAGTACTGGGT
9	TGTCCTTCAGCCGCGCGGCTCCAATCAGCAGCGAGTACTGGGT
10	TGTCCTTCAGCCGCGCGGCTCCACTCAGCAGCGAGTACTGGGT
11	ACCCAGTACTCGCTGCTGAAGGGAGCCGCGCGGCTGAAGGACA
12	ACCCAGTACTCGCTGCTGAAAGGAGCCGCGCGGCTGAAGGACA
13	ACCCAGTACTCGCTGCTGAACCGAGCCGCGCGGCTGAAGGACA
14	ACCCAGTACTCGCTGCTGAAUGGAGCCGCGCGGCTGAAGGACA
15	TGTCCTTCAGCCGCGCGGCTCCHTTCAGCAGCGAGTACTGGGT
16	CCGTTCAGCAGCGAGTACTGGGT
17	AATGGAGCCGCGCGGCTGAAGGACA

*6FAM labeled oligonucleotide at the 5'-end

Mismatch or marker name	Combination
none	1+3
G/T	1+4
T/T	1+5
T/C	1+6
A/A	1+7
A/C	1+8
G/G	2+9
G/A	2+10
C/C	2+11
G/U	2+12
T/H	1+13
Marker 1 (M1)	3+16
Marker 2 (M2)	4+17

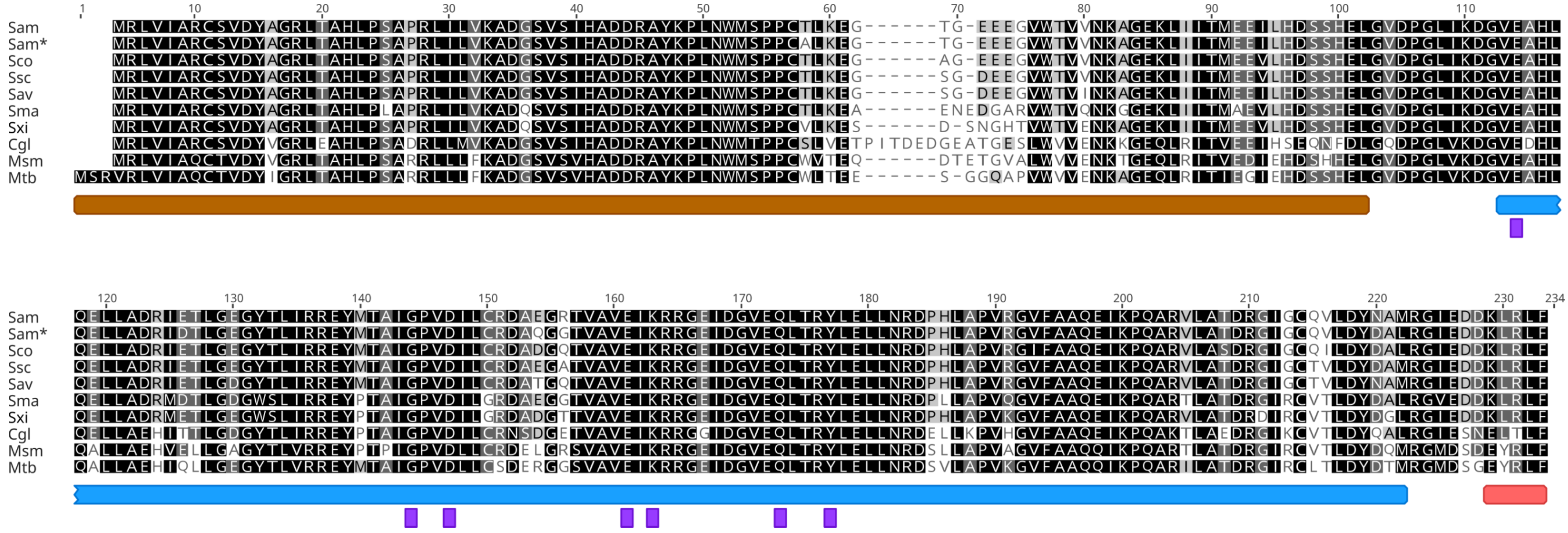

Supplementary Figure S1. Conservation of NucS protein through actinobacteria. Alignment of NucS protein sequences from different species of *Streptomyces* and *Mycobacterium*. *S. ambofaciens* ATCC 28777 (Sam), *S. ambofaciens* DSM 40697 (Sam*), *S. coelicolor* A3(2) (Sco), *S. scabiei* NCPPB 4086 (Ssc), *S. avermitilis* MA 4680 (Sav), *S. xiamenensis* 318 (Sxi), *S. marincola* strain SCSIO 03032 (Sma), *C. glutamicum* ATCC 13032 (Cgl), *M. smegmatis* mc^2 155 (Msm), *M. tuberculosis* H38rv (Mtb). Black, dark grey, light grey and blank boxed residues correspond to 100%, 80-100%, 60-80% and less than 60% sequences presenting the same residue. Colored boxes under the alignment indicate protein domains according to *Pyrococcus abyssi* NucS structure (Ren et al., 2009). DNA-binding and catalytic domains are shown in brown and blue respectively. PIP-box motif is represented in red. Essential residues for nuclease activity are shown in purple.

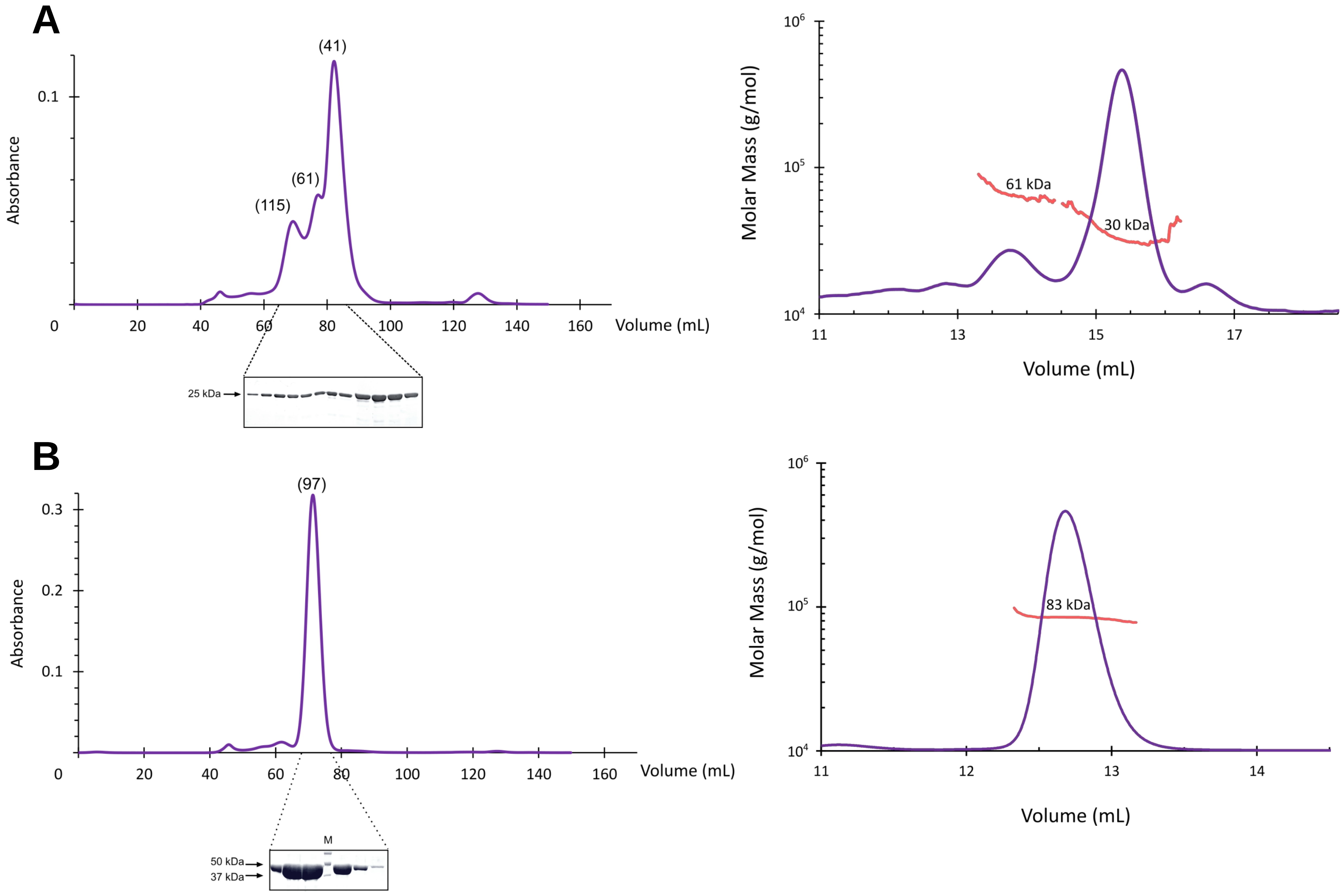

Supplementary Figure S2. Purification and oligomeric states of NucS$_{Sam}$ and β-clamp$_{Sam}$ Gel filtration profile and SEC-MALS analysis (left and right panels, respectively) of (A) NucS$_{Sam}$ and (B) β-clamp$_{Sam}$. For gel filtration, the samples were loaded onto the Superdex 200 16/600 column. Number in brackets correspond to estimated molecular weight (kDa) using calibration curve. Aliquots of the 12 elution fractions from 63 to 87 mL for NucS$_{Sam}$ and the 6 elution fractions from 67 to 79 mL for β-clamp$_{Sam}$ were subjected to 12% SDS-PAGE (lower part). M is the protein ladder "Precision Plus Protein Standard" (Biorad). The SEC-MALS chromatograms display the UV absorbance at 280 nm and red lines indicate the molar mass distribution. Because of the reasonable homogeneity of NucS$_{Sam}$ peaks, the molecular weight was estimated at the right extremity of each peak.

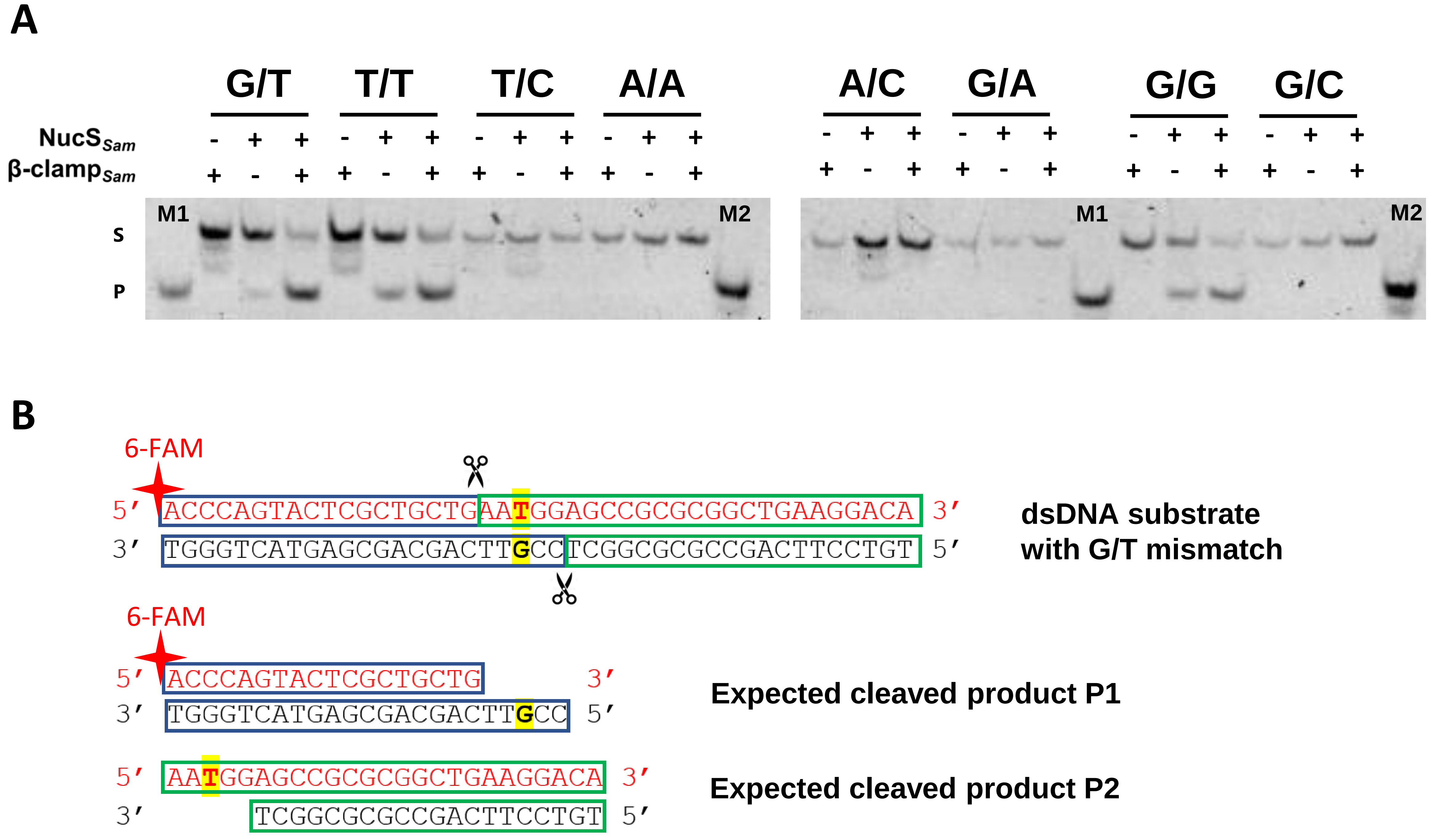

Supplementary Figure S3. Estimation of the size of the cleaved products. (A) *In vitro* cleavage assays were performed with 6-FAM-labeled dsDNA substrates (43-bp, 50 nM) containing no mismatch (control) or single base-pair mismatches (A/A, A/C, C/C, G/A, G/G, G/T, T/C or T/T). Each substrate was incubated for 60 min at 30°C, either with 2.4 µM of NucS$_{Sam}$ or with 1.2 µM of β-clamp$_{Sam}$ or with both proteins at the above concentrations. Products were separated by 10% native PAGE. Substrates and cleavage products are indicated by "S" and "P" letters on the left side of the panel. M1 and M2 DNA markers (see also supplementary Table 2) were used to estimate the products size. (B) DNA products expected after cleavage of a dsDNA substrate containing a G/T mismatch. For G/T substrate, it is the upper strand that is 6-FAM-labeled, therefore only P1 expected product will be detected. Depending on the 6-FAM labeled oligonucleotide used to create the substrate, 2 different DNA markers were used : M1 marker was used to estimate the size of P1 product for G/T, T/T, T/C, A/A, A/C and T/H mismatches while M2 marker was used to estimate the size of P2 product for G/G, G/A, C/C, and G/U mismatches. M1 and M2 markers have been designed with the same composition as P1 and P2 products.

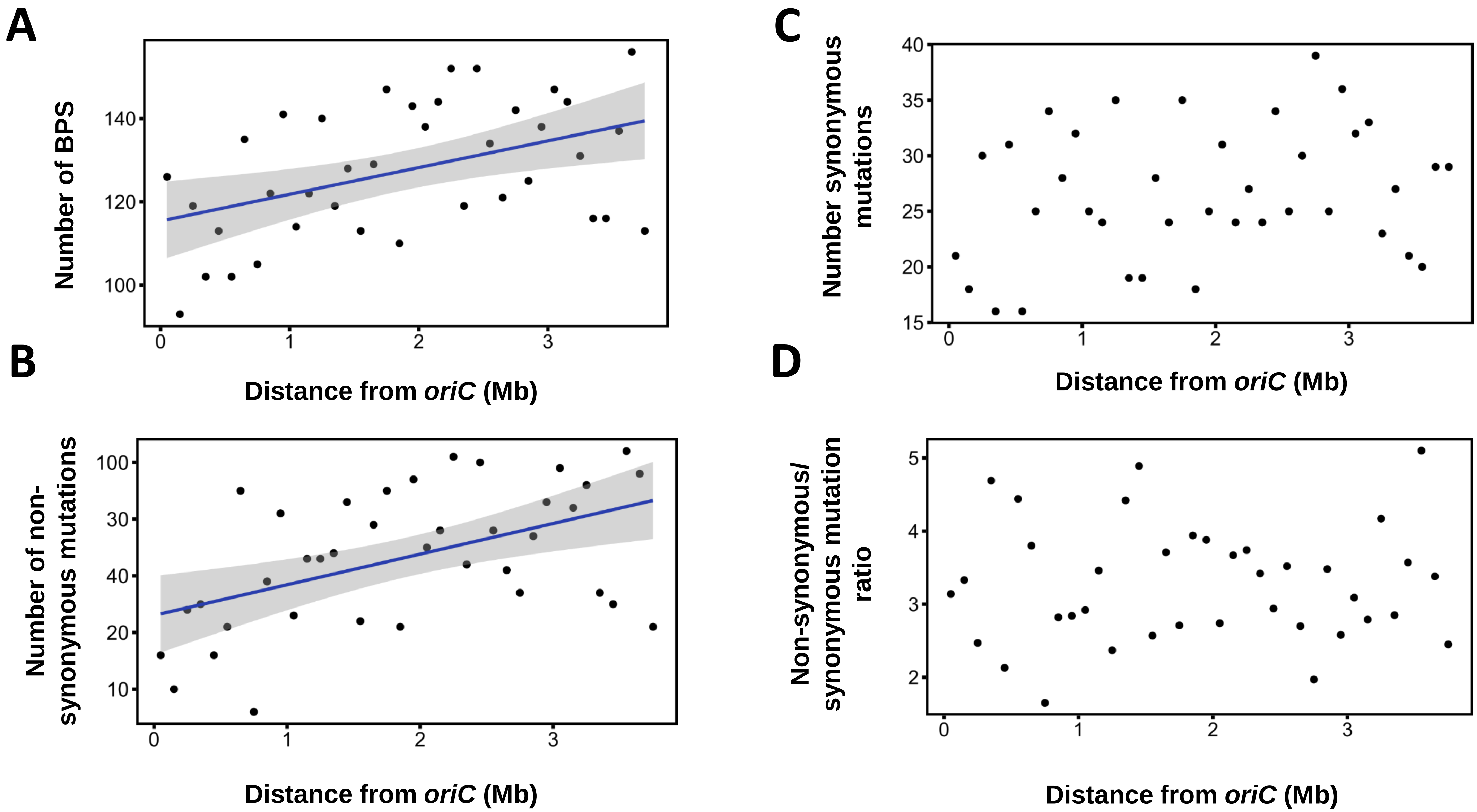

Supplementary Figure S4. Distribution of mutations along the chromosome in Δ*nucS* lines. Base pair substitutions (BPSs), non-synonymous and synonymous mutations within coding sequences, were counted within a non-overlapping 100 kb window, starting from *dnaA* gene (located in the middle of the chromosome at position 4,021,374 and approximately corresponding to the origin of replication, *ori*C) and sliding towards the chromosome extremities. The number of these mutations in windows of the right replichore were added to the number in windows at the same distance of *dnaA* in the left replichore. The sum of BPSs (A), non-synonymous mutations (B), or synonymous mutations (C) is represented as a function of the distance from *dnaA*. The ratio of non synonymous to synonymous mutations (dN/dS) was calculated for each 100 kb window and plotted as a function of the genomic position (D). Significant positive correlations were observed between the genomic position and the BPS count (Pearson's correlation coefficient r=0.451, P=0.004), or between the genomic position and the non-synonymous mutations (Pearson's correlation coefficient r=0.499, P=0.001). No correlation was noted for synonymous mutations or for dN/dS ratio.

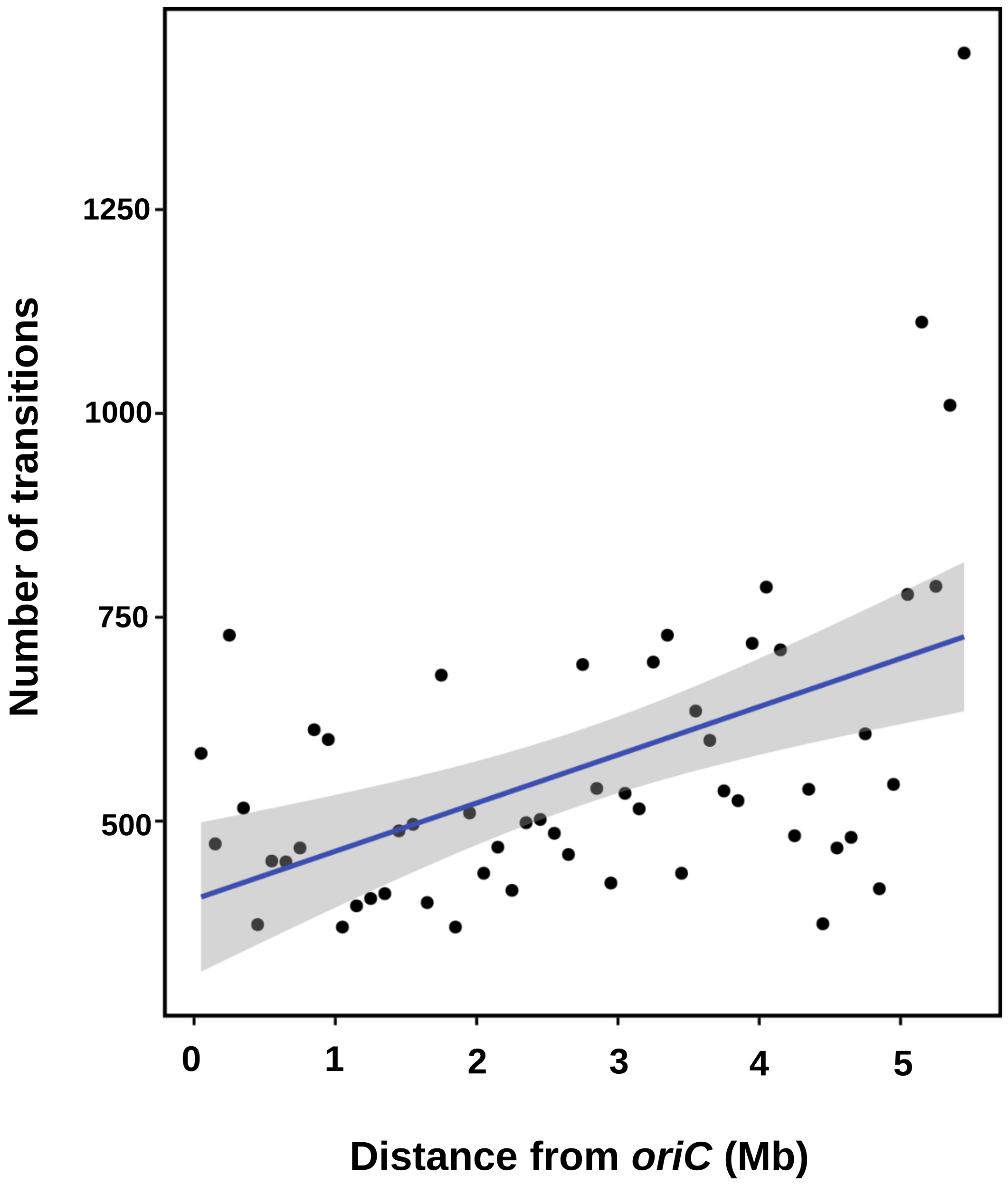

Supplementary Figure S5. Transition distribution along the chromosome of *Streptomyces* environmental strain RLB1-8 compared to *Streptomyces* environmental strain RLB3-17. Transitions were counted within a non-overlapping 100 kb window starting from *dnaA* gene (corresponding to *oriC*, approximately at position 6,204,383 in RLB1-8). The number of transitions in windows of the right replichore was added to the number of transitions of windows at the same distance of *dnaA* in the left replichore , and represented as a function of the distance from *dnaA* gene. A significant positive correlation was observed between the transition count and the genomic position (Kendall rank correlation test *r*=0,33 *P*=0,00045).

Supplementary Table S3. Distribution of BPSs in the replichores

	WT		Δ*nucS*	
	Left Replichore	Right Replichore	Left Replichore	Right Replichore
**Transitions**	**30**	**43**	**2357**	**2551**
A:T>G:C	11	21	1424	1562
A -> G	3	14	393	1150
T -> C	8	7	1031	412
G:C>A:T	19	22	933	989
G -> A	16	12	699	274
C -> T	3	10	234	715
**Transversions**	**36**	**38**	**47**	**93**
A:T>T:A	1	4	1	11
A -> T	1	2	0	7
T -> A	0	2	1	4
A:T>C:G	2	4	8	12
A -> C	1	1	7	2
T -> G	1	3	1	10
G:C>T:A	12	6	14	18
G -> T	11	2	10	5
C -> A	1	4	4	13
G:C>C:G	21	24	24	52
G -> C	11	2	9	29
C -> G	10	22	15	23
**Total**	**66**	**81**	**2404**	**2644**
